# Hindi CCGbank: A CCG treebank from the Hindi dependency treebank

**DOI:** 10.1007/s10579-017-9379-6

**Published:** 2017-01-25

**Authors:** Bharat Ram Ambati, Tejaswini Deoskar, Mark Steedman

**Affiliations:** 1 ILCC, School of Informatics, University of Edinburgh, Edinburgh, UK; 2 Institute for Logic, Language and Computation, University of Amsterdam, Amsterdam, The Netherlands

**Keywords:** Combinatory categorial grammar, CCG, Treebank, Hindi, Non-projective dependencies

## Abstract

In this paper, we present an approach for automatically creating a combinatory categorial grammar (CCG) treebank from a dependency treebank for the subject–object–verb language Hindi. Rather than a direct conversion from dependency trees to CCG trees, we propose a two stage approach: a language independent generic algorithm first extracts a CCG lexicon from the dependency treebank. An exhaustive CCG parser then creates a treebank of CCG derivations. We also discuss special cases of this generic algorithm to handle linguistic phenomena specific to Hindi. In doing so we extract different constructions with long-range dependencies like coordinate constructions and non-projective dependencies resulting from constructions like relative clauses, noun elaboration and verbal modifiers.

## Introduction

Combinatory categorial grammar (CCG) (Steedman 2000) is an efficiently parseable, yet linguistically expressive grammar formalism. In addition to predicate-argument structure, CCG elegantly captures the unbounded dependencies found in grammatical constructions like relativization, coordination etc. Availability of the English CCGbank (Hockenmaier and Steedman 2007) has enabled the creation of several robust and accurate wide-coverage CCG parsers for English, both graph-based and transition-based, that are being used extensively for broad-coverage parsing, and especially for tasks requiring deep linguistic analysis such as semantic parsing and question-answering (Hockenmaier and Steedman 2002; Clark and Curran 2007; Auli and Lopez 2011; Lewis and Steedman 2014; Zhang and Clark 2011; Xu et al. 2014; Ambati et al. 2015). Creation of CCGbanks in other languages, especially languages typologically far from English is beneficial both for the development of CCG analyses for linguistic phenomenon in these languages, and also for the development of deep NLP tools for these languages.

Different grammar formalisms like phrase structure grammar, combinatory categorial grammar, and dependency grammar have different advantages. But developing treebanks manually in each formalism is a very expensive and time consuming task. Automatic conversion of treebanks from one formalism to another significantly reduces the manual annotation effort. We develop an algorithm for automatically creating CCGbanks from dependency treebanks. We apply this approach to automatically creating a Hindi CCGbank from an existing manually created Hindi dependency treebank (Bhatt et al. 2009). The approach is applicable for creating CCGbanks for other languages with existing dependency treebanks, and is especially relevant for other Indian languages.

As compared to English, many Indian languages, including Hindi, while basically verb final, have a freer word-order and are morphologically richer. All of these characteristics pose challenges to statistical parsers. In the Hindi dependency treebank there are around 20% of dependency trees with at least one non-projective arc which are problematic for vanilla shift-reduce parsing algorithms like arc-eager and arc-standard (Nivre et al. 2007b). In this work, we show that CCG can capture these phenomena elegantly, essentially by making such dependencies projective—that is, covered by the grammar. Our approach can be adapted to extract CCGbanks for other typologically similar languages with existing dependency treebanks, such as other Indic languages. The rest of the paper is organized as follows. Section 2 gives a short introduction to the CCG formalism. Section 3 describes related work regarding the automatic creation of CCGbanks for English and other languages. A brief summary of the Hindi dependency treebank is provided in Sect. 4. In Sects. 5 and 6, we first show how we extract a CCG lexicon from the Hindi dependency treebank and then use it to create a Hindi CCGbank. Details of different long-range dependencies arising from coordination and other non-projective constructions are presented in Sects. 7 and 8. Finally, an analysis of CCG categories and combinators present in the Hindi CCGbank is provided in Sect. 9. We conclude with possible future directions in Sect. 10.

## Combinatory categorial grammar

Combinatory categorial grammar (CCG) is a strongly lexicalized grammar formalism, in the sense that all language-specific information including linear order is defined at the level of the lexicon. It is “nearly context-free” in expressive power, in the sense of being among a group of formalisms for natural language grammars that are at the lowest level of the language hierarchy above context-free grammar (CFG) that is known (Joshi et al. 1991; Kuhlmann et al. 2015). It has a completely type-transparent interface between syntactic derivation and compositional assembly of the underlying semantic representation, including predicate-argument structure, quantification and information structure. Because of this semantic transparency, CCG is widely used in practical applications involving semantic interpretation and inference, (Bos et al. 2004; Lewis and Steedman 2013a, b) especially for semantic parsing with special focus on question answering (Kwiatkowski et al. 2013; Reddy et al. 2014).

In the categorial lexicon, words are associated with syntactic categories, such as $S\setminus NP$ or $(S\setminus NP)/ NP$ for English intransitive and transitive verbs. Categories of the form $X\setminus Y$ or *X* / *Y* are functors, which take an argument *Y* to their left or right (depending on the direction of the slash) and yield a result *X*. Every syntactic category is paired with a semantic interpretation (usually expressed as a $\lambda$-term).

Like all variants of categorial grammar, CCG uses function application to combine constituents, but it also uses a set of linear order-dependent syntactic combinatory rules corresponding semantically to composition (**B**) and type-raising (**T**). Type raising is a non-recursive lexical operation related to (morphological or “structural”’) case. However, for fixed word-order languages without morphological case, Hockenmaier and Steedman (2007) advocate the use of unary type-changing? rules for reasons of efficiency, including type-raising rules and additional rules to deal with complex adjunct categories (e.g $(NP\setminus NP)\Longrightarrow S[ng]\setminus NP$ for ing-VPs that act as noun phrase modifiers). Examples of CCG combinators are:

**Table:** 

Forward Application (>)	X/Y	Y	$\Longrightarrow$	X
Backward Application (<)	Y	$\hbox {X}\setminus \hbox {Y}$	$\Longrightarrow$	X
Forward Composition (>B)	X/Y	Y/Z	$\Longrightarrow$	X/Z
Backward Composition (<B)	$\hbox {Y}\setminus \hbox {Z}$	$\hbox {X}\setminus \hbox {Y}$	$\Longrightarrow$	$\hbox {X}\setminus \hbox {Z}$
Forward Crossed Composition (${>}B_{X}$)	X/Y	$\hbox {Y}\setminus \hbox {Z}$	$\Longrightarrow$	$\hbox {X}\setminus \hbox {Z}$
Backward Crossed Composition (${<}B_{X}$)	Y/Z	$\hbox {X}\setminus \hbox {Y}$	$\Longrightarrow$	X/Z
Forward Type-raising (>T)	X		$\Longrightarrow$	$\hbox {T}/(\hbox {T}\setminus \hbox {X})$
Backward Type-raising (<T)	X		$\Longrightarrow$	$\hbox {T}\setminus (\hbox {T/X})$

## Related work

Hockenmaier and Steedman (2007) developed the first English CCGbank automatically from the Penn Wall Street Journal Phrase Structure Treebank (Marcus et al. 1993). For each phrase structure tree, they first determine the constituent type of each node using heuristics adapted from Magerman (1994) and Collins (1999), which take the label of a node and its parent into account. Then the tree is binarized inserting dummy nodes as required into the tree such that all children to the left of the head branch off in a right-branching tree, and then all children to the right of the head branch off in a left-branching tree. Then CCG categories are assigned based on whether the node is root of the sentence, complement or adjunct of the head. Finally, headword dependencies which approximate the underlying predicate-argument structure are obtained.

The English CCGbank (Hockenmaier and Steedman 2007) is primarily created from the Penn Phrase Structure Treebank, which doesn’t directly capture interesting linguistic phenomena like predicate-argument structures. Resources like PropBank (Palmer et al. 2005) capture predicate-argument structure of the verb. Using PropBank, Honnibal and Curran (2007) improved the complement and adjunct distinction in the CCGbank. Using information from different resources like PropBank and NomBank (Meyers et al. 2004), Honnibal et al. (2010) created an updated version of CCGbank which includes predicate-argument structures for both verbs and nouns, baseNP brackets, verb-particle constructions, and nominal modifiers. They also trained a state-of-the-art CCG parser on this new treebank and compared with the original treebank. Since the updated treebank contains fine-grained details the performance of the parser was slightly lower than the one trained on the original version.

Following Hockenmaier and Steedman (2007), there have been some efforts at automatically extracting treebanks of CCG derivations for other languages. Hockenmaier (2006) developed a CCGbank for German from the Tiger treebank (Brants et al. 2002). The Tiger treebank is based on a framework which has features from both phrase structure grammar and dependency grammar and results in graphs rather than trees. First, these graphs are pre-processed and converted to planar trees. Then a translation step is applied which binarizes the planar tree and extracts the CCG derivation. Tse and Curran (2010) use an algorithm similar to Hockenmaier and Steedman (2007) to extract a Chinese CCGbank from the Penn Chinese Treebank (Xue et al. 2005).

There has also been work on extracting CCG lexicons (Cakici 2005) and CCGbanks (Bos et al. 2009; Uematsu et al. 2013, 2015) from dependency treebanks. Bos et al. (2009) created an Italian CCGbank from the Turin University Treebank (TUT),^1^ an Italian dependency treebank. They first converted dependency trees into phrase structure trees and then applying an algorithm similar to Hockenmaier and Steedman (2007) extracted the CCG derivations. Using different dependency resources available for Japanese like the Kyoto corpus (Kawahara et al. 2002) and the NAIST text corpus (Iida et al. 2007), Uematsu et al. (2013) developed a CCGbank for Japanese. They first integrated the dependency resources into phrase structure trees and then converted them into CCG derivations.

Cakici (2005) extracted a CCG lexicon for Turkish. She first made a list of complement and adjunct dependency labels. Traversing the dependency tree, she assigned CCG categories to each node based on complement or adjunct information. Following Cakici (2005), we first extract a Hindi CCG lexicon from the dependency treebank. Then we use a CKY parser based on the CCG formalism to automatically obtain a treebank of CCG derivations from this lexicon, a novel methodology that may be applicable to obtaining CCG treebanks in other languages as well. Our algorithm for extracting the lexicon is similar to Cakici (2005), but with pre-processing steps specific to Hindi. However, where Cakici (2005) extracted only a CCG lexicon, we extended it by developing a novel methodology for creating CCG derivations from this lexicon. Kumari and Rao (2015) have successfully applied our method to create a CCGbank for Telugu, an Indian language, differing from Hindi in belonging to the Dravidian language family, and being agglutinative, suggesting that our algorithm is generic enough to be applied to other languages with little effort.

In this paper, we first explain the process of creating a Hindi CCGbank from the dependency treebank using the approach described in Ambati et al. (2013). Then we consider long-range dependencies in coordination constructions and other so called non-projective constructions and show how they can be handled within the extended form of syntactic projection afforded by CCG.

## Hindi dependency treebank

In this section, we first give a brief introduction to the Hindi language. Then we provide details about the Paninian grammatical model used for Hindi dependency annotation. Following this, we describe the Hindi dependency treebank.

### Hindi language

Hindi is one of the official languages of the Republic of India, and the 4th largest language in the world, with over 260 million speakers.^2^ Hindi, while basically verb final, is a freer word-order language. This can be seen in (1), where (1a) shows the constituents in the default SOV (Subject, Object, Verb) order, and the remaining examples show some of the word-order variants of (1a).^3^

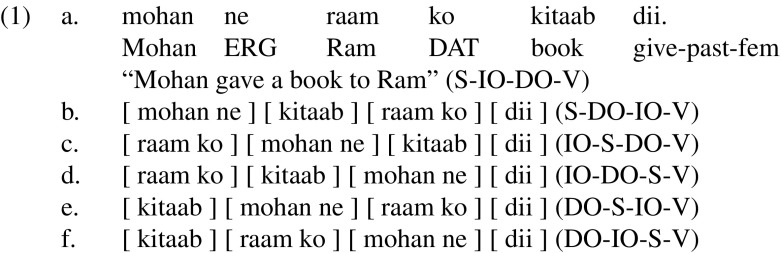



Hindi also has a rich case marking system, although case marking is not obligatory. For example, in (1), while the subject and indirect object are explicitly marked for the ergative^4^ (ERG) and dative (DAT) cases, the direct object is unmarked for the accusative.

### Paninian grammatical model

Indian Languages (ILs) including Hindi are morphologically rich and have a relatively flexible word-order. For such languages, the syntactic notions of subject and object are not able to explain the varied linguistic phenomena. In fact, there is a debate in the literature whether the notions ‘subject’ and ‘object’ can at all be defined for ILs (Mohanan 1982). Behavioural properties are the only criteria based on which one can confidently identify grammatical functions in Hindi (Mohanan 1994); it can be difficult to exploit such properties computationally. Marking semantic properties such as thematic role as dependency relation is also problematic. Thematic roles are abstract notions and will require higher semantic features which are difficult to formulate and to extract as well. The Paninian grammatical model (Kiparsky and Staal 1969; Shastri 1973) provides a level which while being syntactically grounded also helps in capturing semantics. In this section we briefly discuss the Paninian grammatical model for ILs and lay down some basic concepts inherent to this framework.

The Paninian framework considers information as central to the study of language. When a writer/speaker uses language to convey some information to the reader/ hearer, he/she codes the information in the language string. Similarly, when a reader/ hearer receives a language string, he/she extracts the information coded in it. The Paninian grammatical model is primarily concerned with: (a) how the information is coded and (b) how it can be extracted.

Two levels of representation can be readily understood in language: One, the actual language string (or sentence), two, what the speaker has in his mind. The latter can also be called as the meaning. Paninian framework has two other important levels: karaka level and vibhakti level

**Fig. 1:**
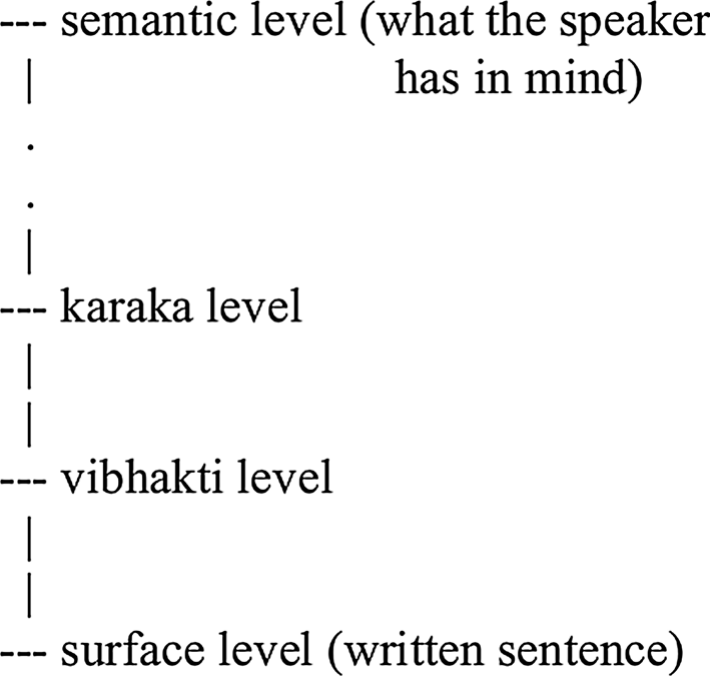
Levels of representation/analysis in the Paninian model

The surface level is the uttered or written sentence. The vibhakti level is the level at which there are local word groups together with case endings, preposition or postposition markers. The vibhakti level abstracts away from many minor (including orthographic and idiosyncratic) differences among languages. Above the vibhakti level is the ‘karaka’ level. It includes karaka relations, which are syntactico-semantic relations between a predicate and its arguments, and a few additional relations such as purpose. The topmost level relates to what the speaker has in his mind. This may be considered to be the ultimate meaning level that the speaker wants to convey. One can imagine several levels between the karaka and the ultimate level, each containing more semantic information. Thus, the karaka level is one in a series of levels, but one which has relationship to semantics on the one hand and syntax on the other. The levels of representation in the Paninian model are presented in Fig. 1.

At the karaka level, we have karaka relations and verb-verb relations, etc. Karaka relations are syntactico-semantic relations between the verbs and other related constituents (typically nouns) in a sentence. They capture a certain level of semantics which is somewhat similar to thematic relations but different from it (Bharati et al. 1995). This is the level of semantics that is important syntactically and is reflected in the surface form of the sentence(s). Begum et al. (2008b) have subsequently proposed and developed an annotation scheme for a dependency treebank based on the Paninian framework. They have extended the original formulation to account for previously unhandled syntactic phenomenon.

The Paninian approach treats a sentence as a set of modifier-modified relations. A sentence is supposed to have a primary modifiee which is generally the main verb of the sentence. The elements modifying the verb participate in the action specified by the verb. The participant relations with the verb are called karaka. The notion of karaka will incorporate the ‘local’ semantics of the verb in a sentence, while also taking cue from the surface level morpho-syntactic information (Vaidya et al. 2009). There are six basic karakas, namely;

- k1: karta (This is similar to subject or agent): the most independent participant in the action
- k2: karma (roughly the theme or object): the one most desired by the karta
- k3: karana (instrument): which is most essential for the action to take place
- k4: sampradaan (beneficiary): recipient or beneficiary of the action
- k5: apaadaan (source): movement away or separation from a source
- k7: adhikarana (location): location of the action in time and space

From the above description, it is easy to see that this analysis is a dependency based analysis (Kiparsky and Staal 1969; Shastri 1973), with the verb as the root of the tree along with its argument structure as its children. The labels on the edges between a child-parent pair show the relationship between them. In addition to the above six labels many others have been proposed as part of the overall framework (Begum et al. 2008b; Bharati et al. 2009). “Appendix 1” shows the most frequent dependency labels with their English equivalent. In this paper we use English labels rather than the Paninian.

In the following section, we provide details of the treebank annotated for Hindi using this Paninian grammatical model.

### Treebank

**Fig. 2:**
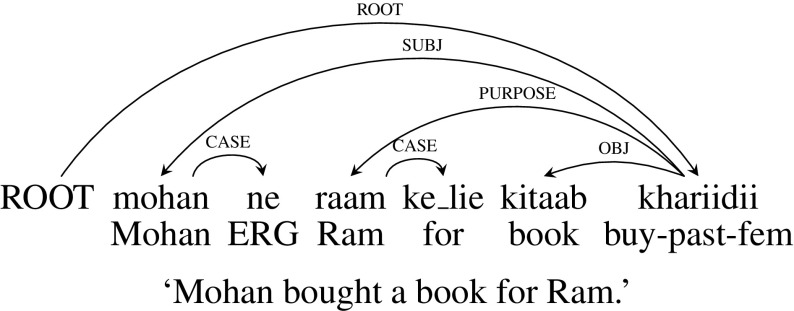
An example dependency tree for Hindi (*ERG* ergative case)

In this work, we consider a subset of the Hindi Dependency Treebank (HDT ver-0.5) released as part of Coling 2012 Shared Task on parsing (Bharati et al. 2012). HDT is a multi-layered dependency treebank (Bhatt et al. 2009) annotated with morpho-syntactic (morphological, part-of-speech and chunk information) and syntactico semantic (dependency) information (Bharati et al. 2006, 2009). POS and chunk information is annotated following the POS and chunk annotation guidelines (Bharati et al. 2006). The morphological features have eight mandatory feature attributes for each node. These features are classified as root, coarse POS category, gender, number, person, case, post position (for a noun) or tense aspect modality (for a verb) and suffix. The dependency annotation follows the Paninian grammar scheme described in Sect. 4.2 which is known to be well-suited to modern Indian languages. Dependency labels are fine-grained, and mark dependencies that are syntactico-semantic in nature, such as agent (usually corresponding to subject), patient (object), and time and place expressions. There are special labels to mark long distance relations like relative clauses, coordination etc (Bharati et al. 1995, 2009). Figure 2 presents the dependency tree for an example sentence *mohan ne raam ke lie kitaab khariidi* (“Mohan bought a book for Ram”).^5^ For readability reasons, we will refer to dependency labels with their English equivalents (e.g., subj, obj, purpose, case for k1, k2, rt, lwg__psp respectively). A list of the Hindi dependency labels and their English equivalents are provided in the “Appendix 1”.

In this example, the verb *khariidii* (“bought”) is the root of the sentence. *mohan* (“Mohan”) is the subject (SUBJ) of the verb *khariidii* (“bought”) and *kitaab* (“book”) is the object (OBJ) of the verb. Since the book is bought for *raam* (“Ram”), *raam* is attached to the verb with PURPOSE dependency label. The post-position markers *ne* (Ergative case marker) and *ke_lie* (equivalent to preposition “for”) are attached to corresponding nouns with CASE dependency label.

The Hindi dependency treebank contains 12,041 training, 1233 development and 1828 testing sentences with an average of 22 words per sentence. Data is provided in the Shakti Standard Format (Bharati et al. 2007) and CoNLL format. The CoNLL format contains word, lemma, pos-tag, and coarse pos-tag in the word, lemma, pos, and cpos fields respectively and morphological features, and chunk information in the feats column.^6^ We use CoNLL format for all our experiments.

## Extracting a CCG lexicon

In order to assign CCG lexical categories to words in the treebank sentences, we first make a list of argument and adjunct dependency labels in the treebank. We obtained this list from the Hindi verb frames which make a distinction between arguments and adjuncts for different verbs, from Begum et al. (2008a). For e.g., dependencies with the label SUBJ and OBJ (corresponding to subject and object respectively) are considered to be arguments, while labels like PLACE and TIME (corresponding to place and time expressions) are considered to be adjuncts.

Starting from the root of the dependency tree, we traverse each node. The category of a node depends on both its parent and children. If the node is an argument of its parent, we assign the chunk tag of the node (e.g., NP, PP) as its CCG category. Otherwise, we assign it a category of *X*|*X*, where *X* is the parent’s *result* category and | is directionality ($\backslash$ or  / ), which depends on the position of the node w.r.t. its parent. The *result* category of a node is the category obtained once its argument slots are saturated. For example, $S_{f}$, is the result category for $(S_{f}\setminus NP)\setminus NP$. Once we get the partial category of a node based on the node’s parent information, we traverse through the children of the node. If a child is an argument, we add that child’s chunk tag, with appropriate directionality, to the node’s category. If the child is an adjunct, the category of the node is not effected.

**Fig. 3:**
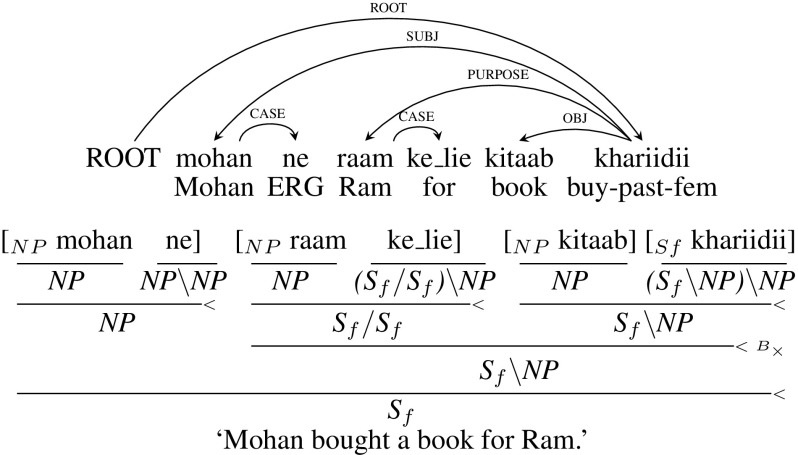
An example dependency tree with its CCG derivation

Consider the verb *khariidii* (“bought”) in the example sentence in Fig. 3. Since it is the root of the sentence which is an argument dependency label, it gets a category $S_{f}$, from its parent. It has three children *mohan* (“Mohan”), *raam* (“Ram”) and *kitaab* (“book”). We traverse through each child and update the category of *khariidii* as follows. *Mohan* is subject (“SUBJ”) of *khariidii*. Since SUBJ is a mandatory argument, the category of *khariidii* is updated to $S_{f}\setminus NP$. The dependency label between *raam* and *khariidii* is PURPOSE which is an adjunct label. So, the category of *khariidii* (“bought”) is not changed due to this child. The third and final child *kitaab* is an object (“OBJ”) of the verb, which is an argument label. As a result, the category of *khariidii* is updated to $(S_{f}\setminus NP)\setminus NP$.^7^

Now we consider again the children of the verb *khariidii* (“bought”). *mohan* (“Mohan”) is an argument of *khariidii*, and hence *NP* is the category for this node. *mohan* (“Mohan”) has a case marker *ne* (“ERG”) as a child with the dependency label CASE. The category of *mohan* (“Mohan”) is not changed and remains *NP*. Now consider the child of *mohan* (“Mohan”) which is *ne* (“ERG”). Since *NP* is the result category of its parent *mohan* (“Mohan”) on the left, category of *ne* (“ERG”) will be ${NP\setminus NP}$.^8^ Categories of other nodes are assigned similarly.

**Fig. 4:**
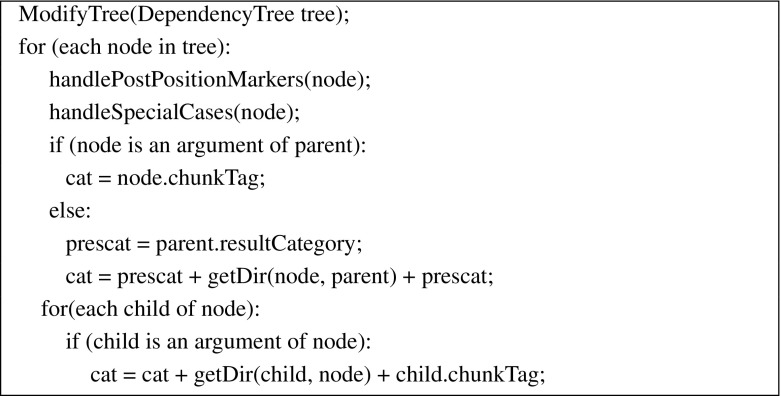
Algorithm for extracting a CCG lexicon from a dependency tree

The algorithm is sketched in Fig. 4 and an example of a CCG derivation for a simple sentence, marked with chunk tags, is shown in Fig. 3. NP and S$_{f}$ are the chunk tags for noun and finite verb chunks respectively.^9^ Some important special cases are described in detail in the following subsections.

The process described above yields a “coarse-grained” lexicon, in which case is not distinguished. We also created a “fine-grained” lexicon, in which we retain morphological information in noun categories. For example, consider the noun chunk *raam ne* (“Ram ERG”). In the fine-grained lexicon, the CCG categories for *raam* and *ne* are *NP* and ${NP[ne]\setminus NP}$ respectively. Morphological information such as ergative case ‘-ne’ in noun categories is expected to help with determining their dependency labels, but makes the lexicon more sparse. We therefore extract both a coarse-grained and a fine-grained lexicon; details of the machine-readable format for both lexicons is presented in “Appendix 3”.

### Morphological markers

In Hindi, morphological information is encoded in the form of post-positional markers on nouns, and tense, aspect and modality markers on verbs. A post-positional marker following a noun plays the role of a case-marker (e.g., *raam ne* (“Ram ERG”), here *ne* is the ergative case marker) and a role similar to an English preposition (e.g., *mej par* (“table on”), here *par* is the postpositional equivalent of the English preposition “on”). Post-positional markers on nouns can be simple one word expressions like *ne* or *par*, or multiple words as in *raam ke lie* (“Ram for”). Complex post position markers as a whole give information about how the head noun or verb behaves. For example, *ke lie* is equivalent to “for” and *ke baare me* is equivalent to “about”. The Hindi CCGbank merges complex postpositional markers into single words like *ke_lie* so that the entire marker gets a single CCG category.

For the “fine-grained” lexicon, we explored two variants of the lexicon: normal and type-raised. In the normal version, the ergative case marker like *ne* bears a category ${NP[ne]\setminus NP}$, looking for an *NP* to the left to yield the case-marked category *NP*[*ne*]. In the type-raised version, the category of *ne* takes an *NP* to its left and creates a category which looks for a VP category ${S_{f}\setminus NP[ne]}$.

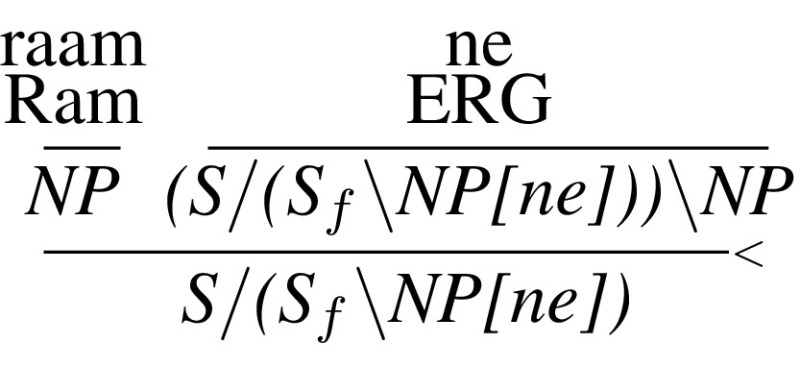



In this variant, the result category ${S/(S_{f}\setminus NP[ne])}$ is the full categorial realization of a Hindi ergative cased *NP* for which *NP*[*ne*] is simply a shorthand.

For an adjunct like *raam ke_lie* (“for Ram”) in Fig. 3, we pass the adjunct information to the post-position marker *ke_lie*, with *NP* as the category for the head noun phrase, and the category ${(S_{f}/ S_{f})\setminus NP}$ for the postposition. Adjuncts that modify adjacent adjuncts are assigned identical categories *X* / *X* making use of CCG’s composition rule and following Cakici (2005).

## CCG lexicon to treebank conversion

Phrase structure to CCG conversion algorithms like Hockenmaier and Steedman (2007) first convert a phrase structure tree into a binary tree. Converting a dependency tree into a binary tree is not possible in the presence of a non-projective arc. For the same reason, direct conversion to CCG trees is not straight-forward. Around 20% of sentences in the Hindi dependency treebank have at least one non-projective arc. In a departure from previous approaches, we therefore use a CCG parser to convert the CCG lexicon to a CCG treebank.

Using the algorithm presented in the previous section, we obtained one CCG category for every word in a sentence. We then run a non-statistical CKY chart parser based on the CCG formalism^10^, which gives CCG derivations based on the lexical categories. This gives multiple derivations for some sentences. We rank these derivations using two criteria. The first criterion is correct recovery of the gold dependencies when the CCG derivation is deterministically mapped back onto a dependency structure. Derivations which lead to gold dependencies are given higher weight. In the second criterion, we prefer derivations which yield intra-chunk dependencies (e.g., verb and auxiliary) prior to inter-chunk (e.g., verb and its arguments). For example, morphological markers (which lead to intra-chunk dependencies) play a crucial role in identifying correct dependencies. Resolving these dependencies first helps the parser in better identification of inter-chunk dependencies such as argument structure of the verb (Ambati 2011). We thus extract the best derivation for each sentence, which is then included in the Hindi CCGbank.

### Evaluation

Coverage of the current conversion algorithm, i.e., the number of sentences for which we got at least one complete derivation using this lexicon is 96%. Disabling crossed composition reduced the coverage by around 10%, showing the importance of this rule for a free word-order language with 20% non-projective sentences. The remaining 4% sentences are either cases where there were inconsistent annotations in the original treebank, or constructions which are currently not handled by our conversion algorithm.

As a second method of evaluating the converted Hindi CCG treebank, we obtained dependencies from the CCG treebank and evaluated them against the gold-standard dependencies in the original dependency treebank. We followed the standard category-indexing procedure of Clark and Curran (2007) for this purpose in order to obtain dependency labels. For example, ${(S\setminus NP{_1})\setminus NP{_2}}$ is the indexed version of the category of ${(S\setminus NP)\setminus NP}$, in which the index 1 marks the subject dependency and 2 marks the object dependency. The indices are not used in the CCG grammar itself, but are important for labeling long-range dependencies in this evaluation.

Following Clark and Curran (2007), we manually indexed the CCG categories which occurred at least 10 times in the treebank data. For the rest of the categories, we assigned default indices. The Hindi CCGbank, (which covers 96% of the sentences in the original dependency treebank), correctly captures 99.1% of the dependencies in the dependency treebank, which is the unlabelled recall. Manually providing indices for all categories would give 100% recall but we leave manual annotation of indices for a future version.

In addition, we performed full manual annotation of 165 sentences with their CCG derivations and compared them with the derivations extracted using our automatic conversion algorithm. Our conversion algorithm failed to provide a derivation for two sentences. Out of these two sentences, the original dependency annotation was wrong for one sentence; correcting the annotation helped the algorithm to handle this sentence. The remaining sentence is the case of argument cluster coordination which is not handled in the current version of the Hindi CCGbank. We also extracted dependencies from these CCG derivations and evaluated with the dependencies in the dependency treebank. We could capture 99.7% (unlabelled recall) of the dependencies present in the dependency treebank. The rest are the cases of less frequent CCG categories where the indices were not manually annotated and are incorrect.

**Fig. 5:**
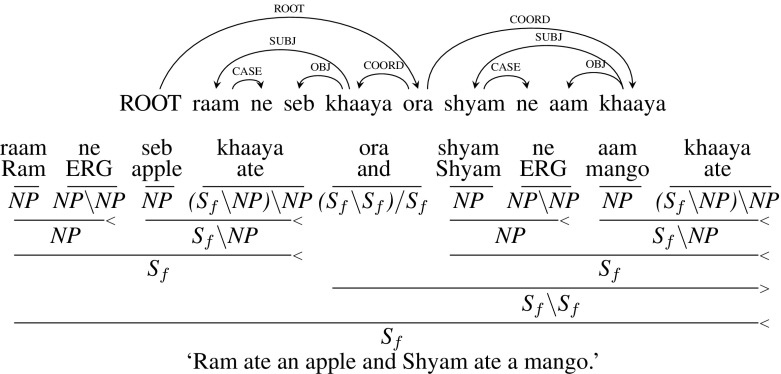
Sentential coordination

## Coordination constructions

Coordination is one of the most frequent sources of long distance dependencies in corpora. Coordination in Hindi can occur between similar components, like noun–noun coordination and verb-verb coordination, but also between some dissimilar but compatible components, like adjective-noun coordination. In the Hindi dependency treebank, there are several instances where an adjectival chunk (JJP) and a noun chunk (NP) are co-ordinated. All of these are cases where the adjectival chunk has an elided noun which is not present explicitly. One such example is

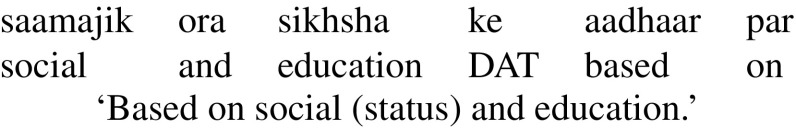



In this example, the coordination is between the adjectival chunk *saamajik* (“social”) and the noun chunk *sikhsha ke* (“education”). The adjectival chunk *saamajik* (“social”) has an elided noun *sthithi* (“status”). When the noun is explicitly present as in *saamajik sthithi* (“social status”) then it is annotated as a noun chunk in the original treebank. But when the noun is not present explicitly, as in *saamajik* (“social”), it is annotated as an adjectival chunk. One can argue for a different annotation scheme and annotate such adjectival chunks as noun chunks. But, for now, to handle these cases, we allowed co-ordination between dissimilar but compatible chunks.

The CCG category of a conjunction is ${(X\setminus X)/ X}$, where a conjunction looks for a child of type *X* to its right and then a child to its left of the same type *X* to yield a result of the same type *X*. Figure 5 gives the dependency tree and CCG derivation for an example sentence with sentential (S) coordination. In the Hindi CCGbank, it is the supertagger that identifies the correct instantiation of the type *X* for the conjunction.^11^

There are four major types of coordination constructions in Hindi. In this section, we first describe each type with an example sentence and then explain how CCG handles them.

**Fig. 6:**
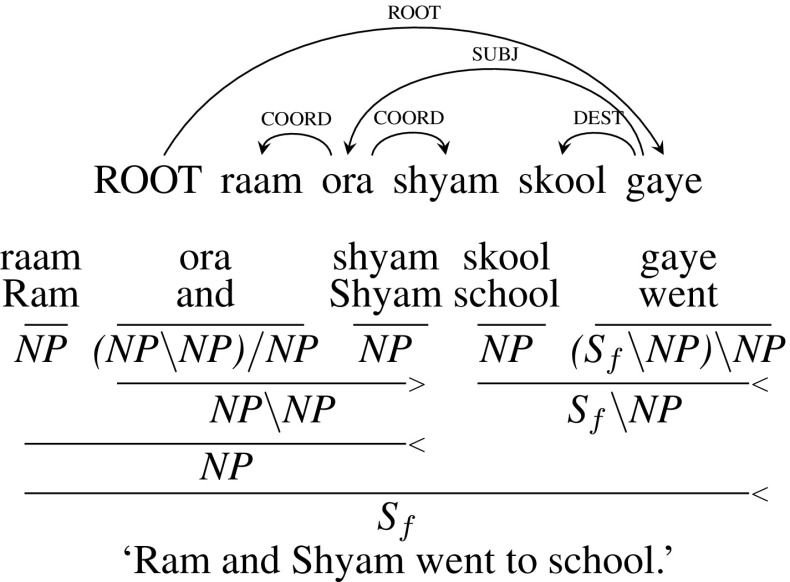
Type 1 coordination

**Type 1** (Conjunction with two children): The CCG category of the conjunction is ${(X\setminus X)/ X}$ where *X* depends on the category of the conjuncts. The example given below in Fig. 6, *raam ora shyam skool gaye* (“Ram and Shyam went to school”), is the case of noun-phrase (NP) coordination. Conjunct *ora* (“and”) has two noun phrases *raam* (“Ram”) and *shyam* (“Shyam”) as its children. Hence the category of *ora* (“and”) is ${(NP\setminus NP)/ NP}$. *ora* (“and”) is first combined with the right child *shyam* and then combined with the left child *raam* leading to a noun phrase, which becomes the subject argument for the verb *gaye* (“went”).

**Fig. 7:**
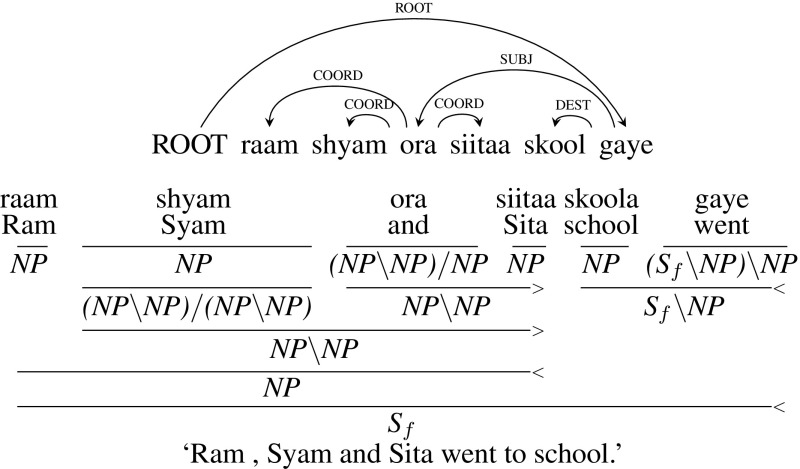
Type 2 coordination

**Type 2** (Conjunction with more than two children and not separated by commas): In Hindi, sometimes a conjunction can have more than two children which are not separated by commas. In such cases, CCG category of the node is type-changed from *X* to a category ${(X\setminus X)/ (X\setminus X)}$. Figure 7 shows the dependency tree of an example sentence *raam shyam ora sita skool gaye* (“Ram Shyam and Sita went to school”). In this example, the conjunct *ora* (“and”) has three children *raam* (“Ram”), *shyam* (“Shyam”) and *sita* (“Sita”). CCG category of *shyam* is type-changed from *NP* to ${(NP\setminus NP)/ (NP\setminus NP)}$ so that it can combine with *ora* and then with *raam* to form an *NP*.

**Fig. 8:**
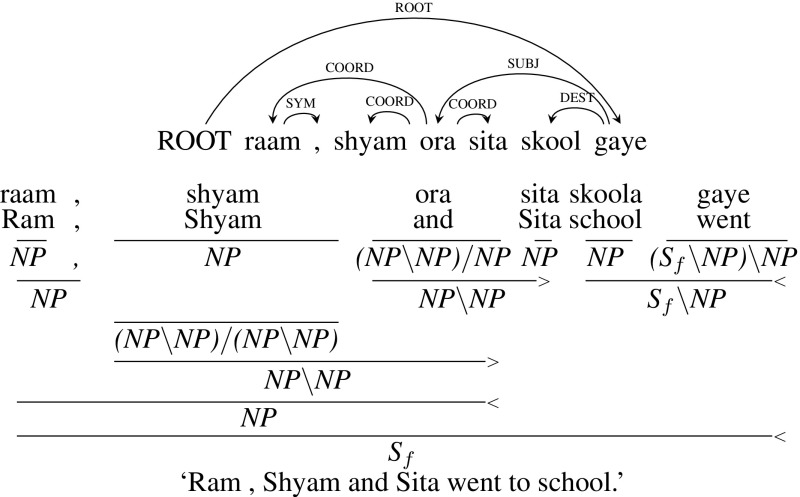
Type 3 coordination

**Type 3** (Conjunction with more than two children separated by commas): The example sentence given below in Fig. 8, *raam , shyam ora sita skool gaye* (“Ram, Shyam and Sita went to school”), is the same as the one presented above in Type 2 category. The only difference is that there is a comma between the nouns *raam* (“Ram”) and *shyam* (“Shyam”). The comma gets a CCG category , which is combined with *NP* to form an *NP*. Similar to Type 2, the CCG category of *shyam* is type-changed from *NP* to ${(NP\setminus NP)/ (NP\setminus NP)}$. This allows *shyam* to combine with *ora* and then with *raam* to form an *NP*.

Unlike other CCGbanks which treat comma as a conjunction, we treat comma as a punctuation here. In that way, we don’t have to change the dependency tree. If we treat a comma as a conjunction, then we have to change the dependency tree as well, where *ora* (“and”) will have comma and *sita* as children and comma will have *raam* and *shyam* as children. Also, since comma can be missing as in Type 2, treating the comma as a punctuation leads to having a single analysis irrespective of whether a comma is present or not.

**Fig. 9:**
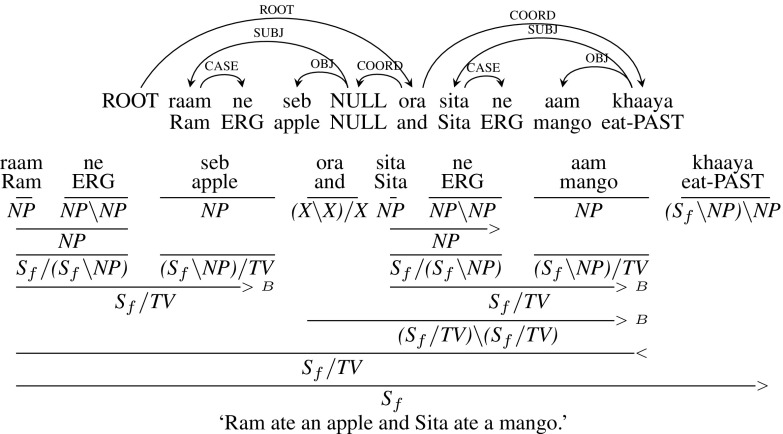
Type 4 coordination

**Type 4** (Argument cluster coordination): Fig. 9 presents an example sentence for argument cluster coordination, *raam ne seb ora sita ne aam khaaya* (“Ram ate an apple and Sita ate a mango”). *khaaya* (“ate”) is the shared verb for both the co-ordinates. To handle such constructions, the dependency tree introduces a dummy “NULL” node which is co-indexed with the main verb *khaaya* and acts as the verb for the 1st sentence as shown in the dependency tree in Fig. 9. CCG can handle such constructions without introducing NULL nodes. The subject *raam ne* is type-raised from *NP* to a category which looks for an intransitive verb, ${S_{f}/ (S_{f}\setminus NP)}$. Similarly, the object *seb* (“apple”) is type-raised from *NP* to a category which looks for a transitive verb, ${(S_{f}\setminus NP)/ TV}$.^12^ Now, these two nodes are combined leading to ${S_{f}/ TV}$ which takes a transitive verb and forms a sentence. Similarly, subject and object arguments of the second sentence, *sita ne* (“Sita”) and *aam* (“Mango”) are type-raised and combined. Now, these type-raised arguments are combined using the conjunction *ora* (“and”) which is then combined with the main verb *khaaya* to form a sentence.^13^

## “Non-projective” constructions

**Fig. 10:**
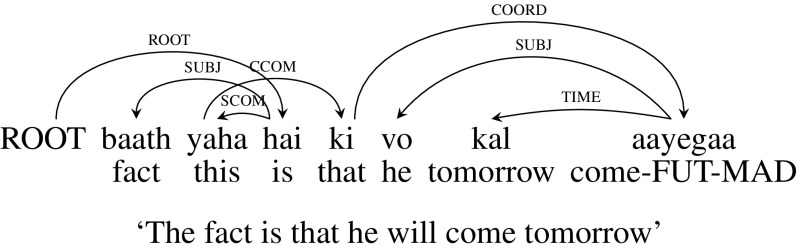
A dependency tree with a “non-projective” dependency

In the tradition of dependency grammar (Hays 1964), constructions which induce dependency arcs which cross as in Fig. 10 are referred to as “non-projective”, because they cannot be generated by the core context-free dependency grammar, and are generally supposed to arise from some separate component of the grammar, such as transformational rules (Robinson 1970).

Such dependencies arise in all languages from processes like relativization and various instances of coordination reduction. To call them “non-projective” is confusing in the present context, since the central claim of CCG is that *all* dependencies are projective, in the sense of arising directly from near-context free syntactic projection. In the dependency parsing literature techniques like swap action (Nivre 2009) or pseudo-projective parsing algorithm (Nivre and Nilsson 2005) are used to handle these crossing arcs. In case of CCG, we can extract such crossing dependencies using indexed categories.^14^ Section 8.3 provides an example derivation showing how indexed categories can be used to extract crossing dependencies. In this section, we present different constructions and/or dependency labels which lead to crossing arcs in the dependency treebank, and explain how CCG can be made to handle them projectively.

Because Hindi has a comparatively free word-order, crossing dependencies are more frequent in the Hindi dependency treebank than in comparable English data. There are a total of 20% sentences with non-projective arcs in the Hindi dependency treebank, amounting to 1.1% of total arcs. There is some previous work on analyzing different non-projective constructions in Hindi and other Indian languages (Mannem et al. 2009; Bhat and Sharma 2012). We categorize the non-projective constructions in the Hindi dependency treebank based on this previous work. Table 1 shows the distribution of non-projective arcs across different constructions.

**Table 1:** Distribution of different non-projective constructions in the treebank

Type of construction	Percentage (%)
Clausal complements	32.4
Relative clause constructions	19.7
Topicalization	15.3
Genitives and dislocated/discontinuous genitives	12.8
Paired connectives	10.5
Others	9.3

In the following sections, we discuss different constructions which lead to crossing arcs in the dependency treebank, and explain how CCG can be made to handle them projectively. In this process, we modified the original dependency tree in two cases: (a) when the original annotation is wrong and (b) in the presence of extraposed clauses. We provide details in the respective sections.

### Clausal complements

**Fig. 11:**
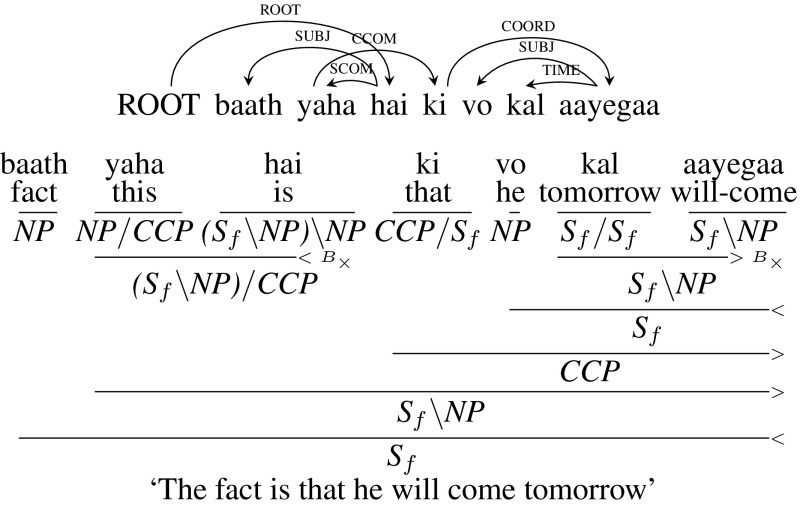
CCOM: CCG derivation (original dependency tree)

Clausal complements of NP forming a complex NP are the cases where clauses elaborate on a noun/pronoun. These are annotated with the CCOM dependency label. For example, in the sentence given below in Fig. 11, *baat* (“fact”) is the subject (“SUBJ”) and *yaha* (“this”) is its noun complement (“SCOM”), which are attached to the verb. Whereas the clause *ki vo kal aayegaa* (“that he will come tomorrow”) has a dependency relation with *yaha* (“this”) and is denoted by CCOM dependency label. 32% of crossing arcs in the treebank are due to this construction.

There are two options to handle this case. In the first option we don’t change the dependency tree. Since *ki* (“that”) is a subordinate conjunction, its chunk tag is CCP. As it looks for a clause/sentence to its right, CCG category for *ki* (“that”) will be ${CCP/ S_{f}}$. This gives *yaha* (“this”) a CCG category of *NP* / *CCP*, since the result category of its child *ki* (“that”) is *CCP*. We can combine *yaha* (“this”) and *hai* (“is”) using Backward Crossing Composition (${<}B_{\times }$) which can then be combined with *ki* (“that”) to establish the crossing dependency. Figure 11 gives the CCG derivation for this example.

**Fig. 12:**
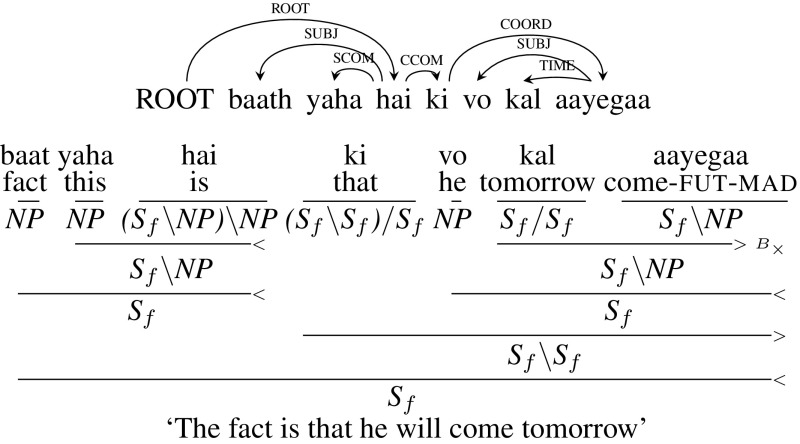
CCOM: CCG derivation (modified dependency tree)

Another option is to systematically change the dependency trees concerned to reflect an analysis in terms of extraposition, where *ki vo kal aayegaa* (“that he will come tomorrow”’) is syntactically a sentential adjunct, and the complement is only linked to its head *baath* (“(the) fact”’) by anaphora at the level of logical form. As a result, the complementizer *ki* is assigned the category $(\mathtt{S}_{f}\setminus \mathtt{S}_{f})/\mathtt{S}_{f}$, which will first combine with the clause to its right *vo kal aayegaa*, and then with the clause to its left *baat yaha hai*, resulting in the derivation shown below in Fig. 12. For the CCGbank conversion, we followed this option and modified the dependency tree so that the CCG derivation is consistent with other extraposed constructions.^15^ We return to the question of extraposition at a number of points below.

### Relative clause constructions

Relative clauses are the second major type of constructions which lead to crossing dependency arcs in the original treebank. 20% of such arcs in the data are due to relative clauses. In the original English CCGbank, relative clauses have the category type ${NP\setminus NP}$, where they combine with a noun phrase on the left to give a resulting noun phrase. Hindi has relative clauses of the type ${NP\setminus NP}$ or *NP* / *NP* based on the position of the relative clause with respect to the head noun.

**Fig. 13:**
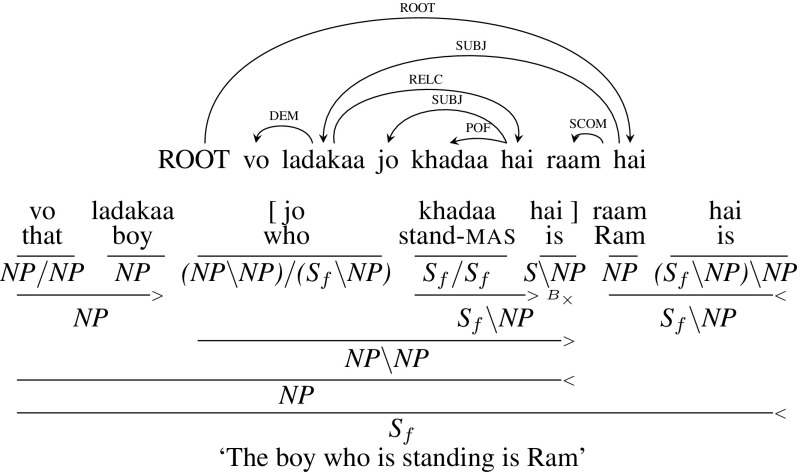
Embedded relative clause

**Fig. 14:**
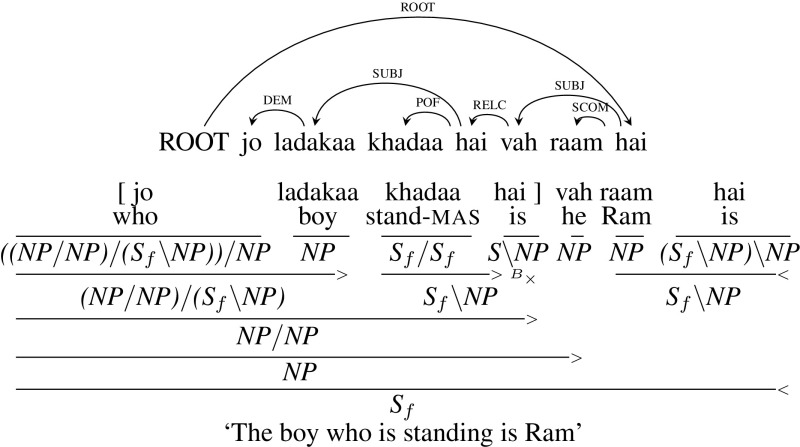
Correlative relative clause

For instance, for the example sentence in Fig. 13, the relative clause has ${NP\setminus NP}$ as its CCG category, since it is to the right of the head noun. Whereas in Fig. 14, the category of the relative clause is *NP* / *NP* since it is to the left of the head noun. Similar to English, in Hindi also, we pass down this information to the relative pronoun rather than the main verb of the relative clause. As a result, the relative pronoun will have a CCG category of (*NP*|*NP*)|*X* where the directionality depends on the position of the relative pronoun in the clause and the category *X* depends on the grammatical role of the relative pronoun.

**Embedded** This is a simple case of relative clause where the relative clause is to the right of its head noun. Mahajan (2000) calls this relative construction as “Normal” since it is similar to the English relative clause construction. This type of relative clause doesn’t lead to crossing dependency arcs. Figure 13 gives an example sentence, *vo ladakaa jo khadaa hai raam hai* (“The boy who is standing is Ram”) with its dependency tree and corresponding CCG derivation.^16^ The relative clause is marked within the brackets in the following figure. In this example, the category of the relative pronoun *jo* (“who”) is ${(NP\setminus NP)/ (S_{f}\setminus NP)}$ which is similar to English relative pronouns. The relative pronoun *jo* (“who”) first combines with the verb phrase *khadaa hai* (“is standing”) to form a relative clause with category ${NP\setminus NP}$. The relative clause then combines with its head noun phrase *vo ladakaa* (“that boy”) which is then combined with the main verb phrase to form a sentence $S_{f}$.

**Correlative** In Hindi, a relative clause can occur to the left of the head noun as well, which is the most frequent form of the construction. This type of relative clause also doesn’t lead to crossing dependency arcs. Figure 14 gives the dependency tree and corresponding CCG derivation for an example sentence, *jo ladakaa khadaa hai vah raam hai* (“The boy who is standing is Ram”). In this example, as the relative pronoun *jo* (“who”) occurs as a demonstrative its category is ${((NP/ NP)/ (S_{f}\setminus NP))/ NP}$. The relative pronoun *jo* (“who”) combines with its head noun *ladakaa* (“boy”) which is then combined with the verb phrase leading to the category of relative clause *NP* / *NP*. Since the relative clause is to the left of the head noun, its category is *NP* / *NP* rather than ${NP\setminus NP}$ which we saw in the previous embedded relative clause.

**Fig. 15:**
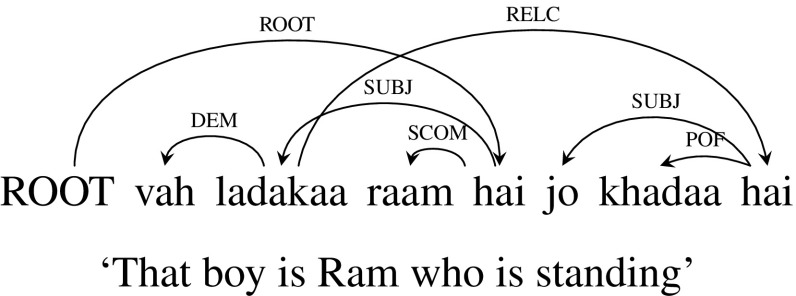
Extraposed relative clause (Example 1): original dependency tree

**Fig. 16:**
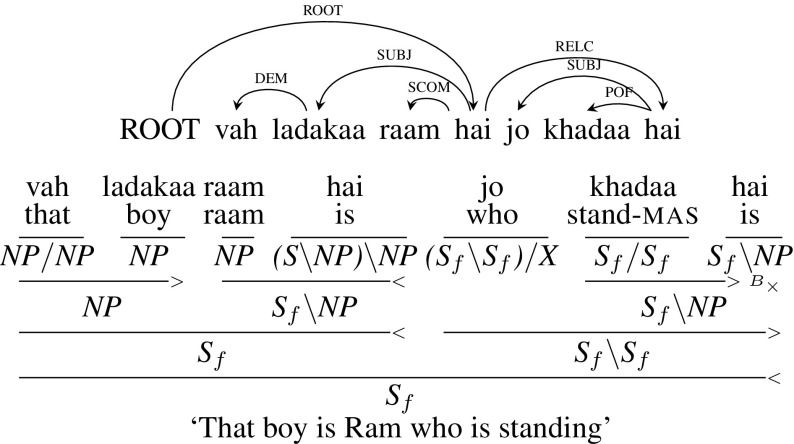
Extraposed relative clause (Example 1): modified dependency tree

**Fig. 17:**
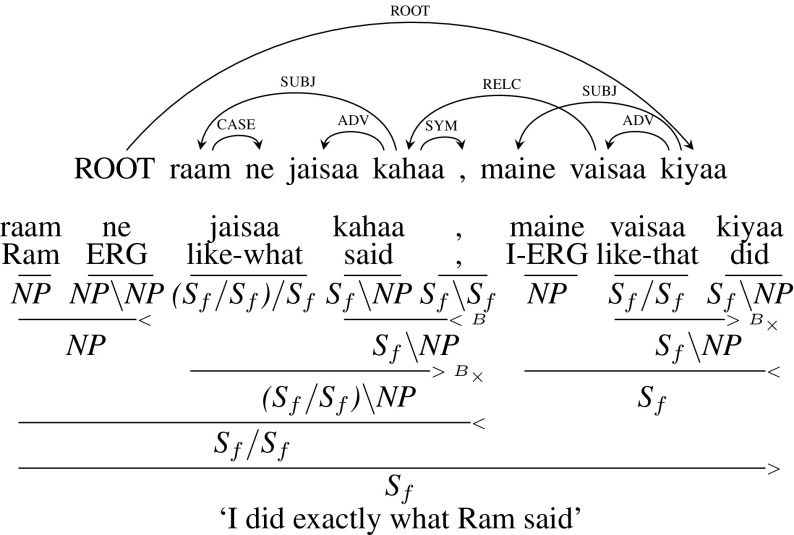
Extraposed relative clause (Example 2)

**Extraposed** Unlike the previous two cases of embedded and correlative constructions where the relative clause is next to the head noun, Hindi, like English, has constructions where the relative clause is not next to its head noun. Figure 15 shows one such example sentence *vah ladakaa raam hai jo khadaa hai* (“That boy is Ram who is standing”). This type of construction lead to a crossing dependency arc. We can’t extract a CCG derivation with the original dependency. Extraposed dependencies are treated anaphorically in CCG, in the semantics, with the extraposed clause treated syntactically as a sentential adjunct. So, to handle this construction, we change the dependency tree slightly. Instead of the relative clause modifying the head noun, we make it modify the main verb. As a result the relative pronoun will have a CCG category of (*S*|*S*)|*X* instead of (*NP*|*NP*)|*X*. Changing the dependency tree is linguistically justified to the extent that extraposed dependencies are generally regarded as not being purely syntactically mediated. Since this is a case of extraposed/dislocated relative clause, the category of relative clause is *S*|*S* rather than *NP*|*NP*. Figure 16 shows the modified dependency tree with corresponding CCG derivation. The problematic RELC arc dependent on the noun *ladakaa* in Fig. 15 is replaced by an arc with the same label dependent on the main verb in Fig. 16. Note that it is easy to recover the dependency between the relative clause and its head noun, as the head noun chunk will have a word whose root is *vo* (“that”).^17^

Figure 17 presents another example sentence which is similar to Fig. 15, except that the relative pronoun is not at the starting of the relative clause and it is also not the mandatory argument of the verb of relative clause. Here, the relative pronoun *jaisaa* (“like-what”) is neither at the beginning of the clause nor a mandatory argument. It is an adverbial modifier (ADV) for the verb *kahaa* (“said”). As a result, the relative pronoun *jaisaa* will have a CCG category ${(S_{f}/ S_{f})/ S_{f}}$. *jaisaa* is combined with the verb *kahaa* (“said”) using forward crossed composition (${B_\times }$) which leads to a category of ${S_{f}/ S_{f}}$ for the relative clause in the end. Similar to the previous example, this is a case of extraposed relative clause.

### Topicalization

The node which is the object/patient of the verb is marked with OBJ dependency label. Topicalization of the object/patient of the verb is the cause for 11.3% of crossing dependency arcs in the treebank.

**Fig. 18:**
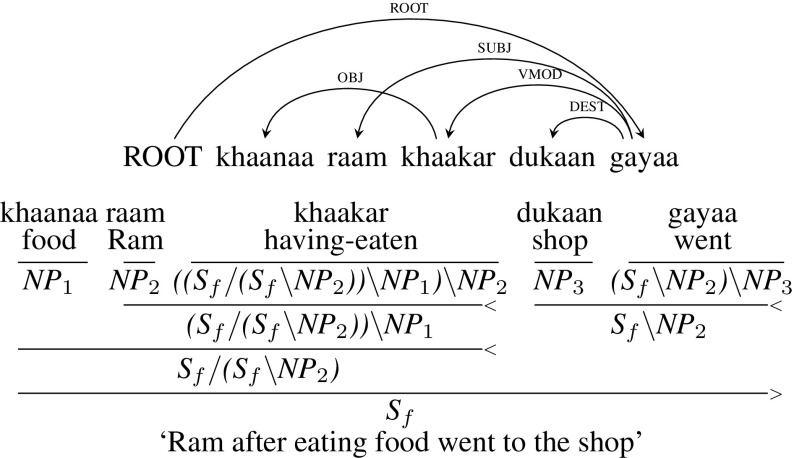
Topicalization

Figure 18 presents an example sentence where a crossing arc is created due to a topicalised object (OBJ) relation. In the example sentence, *khaanaa raam khaakar dukaan gayaa* (“Ram after eating food went to the shop”), there are two verbs: *khaakar* (“having-eaten”), a non-finite verb and *gayaa* (“went”), a finite verb. *raam* (“Ram”) is the shared subject (SUBJ) of both the verbs. As per Hindi dependency guidelines, *raam* cannot have two parents. So it is marked as SUBJ of the main verb *gayaa* (“went”). If the subject, *raam*, was at the start of the sentence then the sentence would be *raam khaanaa khaakar dukaan gayaa*, which is the most frequent construction. Then it would not have created the crossing arc. Shared subject *raam* appearing within non-finite verb phrase *khaanaa khaakar* (“having eaten food”), although grammatical, is not very common in the treebank as compared to the topicalised variant, which is more frequent.

To handle these types of constructions, we relax the constraint of a node having multiple parents. *raam* is subject of both the verbs: *khaakar* (“having eaten”) and *gayaa* (“went”). But due to the tree constraint, the subject *raam* cannot have two parents. We let the CCG derivation have *raam* as the subject for both the verbs. As a result, *khaakar* will have the CCG category ${((S_{f}/ (S_{f}\setminus NP{_2}))\setminus NP{_1})\setminus NP{_2}}$.^18^ The first part of the category, ${(S_{f}/ (S_{f}\setminus NP{_2}))}$, captures the information that it is a verbal modifier which shares an argument with the main verb. *khaakar* (“having-eaten”) first combines with *raam* and then with *khaanaa* (“food”) to form ${S_{f}/ (S_{f}\setminus NP{_2})}$. This is then combined with the VP *dukaan gayaa* (“went to shop”) resulting in a sentence ${S_{f}}$. Note that *gayaa* and *raam* are never combined directly in the derivation. But this dependency is resolved using the indices.

### Paired connectives

Paired connectives such as *agar-to* (“if–then”) are the cause for 10.5% of crossing dependency arcs in the treebank. These constructions involve VMOD, verbal modifier, dependency label. Any verbal modifier which cannot be categorised as a specific relation like subject (SUBJ), object (OBJ) etc. is marked by a VMOD relation.

**Fig. 19:**
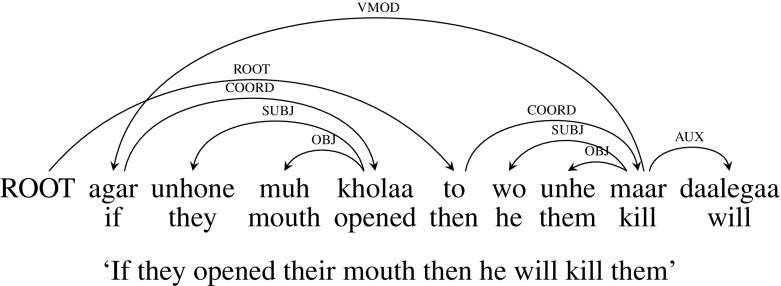
Paired connectives: original dependency tree

**Original Annotation** Figure 19 presents an example ‘if-then’ construction. In the original dependency tree for this sentence, *agar unhone muh kholaa to wo unhe maar daalegaa* (“If they opened their mouth then he will kill them”), *to* (“then”) is the ROOT of the sentence. *maar* (“kill”) is the child of *tho* (“then”) with the dependency relation COORD. *agar* (“if”) is the child of *maar* (“kill”) with dependency relation VMOD and *kholaa* (“opened”) is the child of *agar* (“if”) with dependency relation COORD. VMOD relation between *maar* (“kill”) and *agar* (“if”) leads to a crossing dependency arc here.

**Modified Annotation** We modified the dependency tree to handle this construction since the original dependency annotation is wrong. In the modified tree, *to* (“then”) is still the ROOT of the sentence. Both the verbs *maar* (“kill”) and *kholaa* (“opened”) are children of *to* (“then”) with a COORD dependency relation. *agara* (“if”) is the child of *kholaa* (“opens”) with the dependency relation VMOD.

**Fig. 20:**
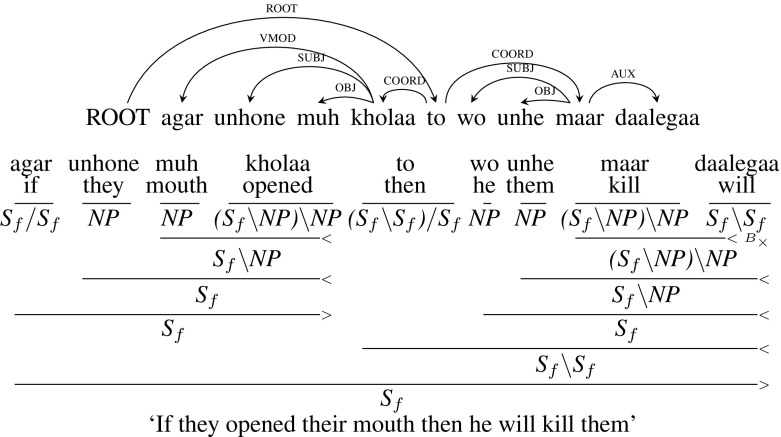
Paired connectives: modified dependency tree and corresponding CCG derivation

In the case of English if-then constructions, the CCG category of *if* is (*S* / *S*) / *S*[*dcl*] which consumes a sentence to its right, leading to an *S* / *S* category for the if-clause. It then consumes the then-clause leading to *S* category. But in the case of Hindi *agar* (“if”) can be optional. To capture this phenomenon, we make the category of *tho* (“then”) to demand *agar* (“if”) clause rather than the opposite. So, the CCG category of *to* (“then”) is ${(S_{f}\setminus S_{f})/ S_{f}}$ which consumes a sentence to its right forming a then-clause with the category ${S_{f}\setminus S_{f}}$. It then combines with a sentence to its left which is the if-clause leading to ${S_{f}}$. Also, as *agar* (“if”) is optional it takes an adjunct category making the main verb the head of the clause. Figure 20 shows the modified dependency tree with the corresponding CCG derivation.

### Genitives and dislocated/discontinuous genitives

**Fig. 21:**
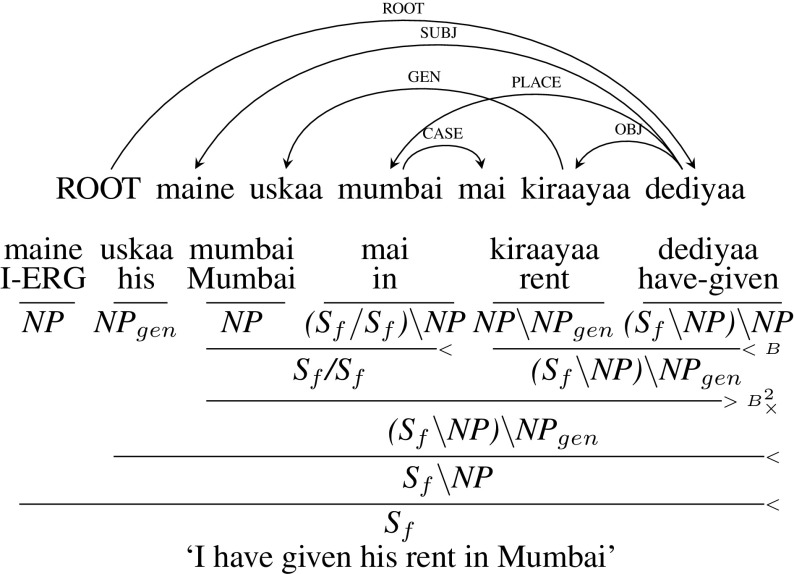
Genitive construction

The genitive/possessive relation which holds between two nouns is marked by GEN dependency label. It mostly occurs with ‘kaa’ (masc.) or ‘kii’ (fem.) postposition marker. A reliable cue for its identification is that the postposition agrees with the noun it modifies in number and gender. In the majority of cases the nouns in genitive relation are next to each other. But, in some cases, due to the free word-order nature of Hindi, some other word can occur between the two nouns in a genitive relation as in the following example in Fig. 21. This construction is the source of 7.5% of the crossing arcs in the the dependency treebank.

In the example in Fig. 21, *maine uskaa mumbai mai kiraayaa dediyaa* (“I have given his rent in Mumbai”), *uskaa* (“his”) and *kiraayaa* (“rent”) are in genitive relation. But, *mumbai mai* (“in Mumbai”) is between these two nouns leading to a crossing arc. Though the dependency labels are different, the construction is similar to the ones described in Sect. 8.5. When two nouns are in a genitive relation, if the both the nouns are next to each other we make the noun with genitive marker demand a noun to its right similar to genitive cases in other languages. But, if both the nouns in genitive relation are not next to each other, then we make the head noun demand the noun with genitive marker as in Fig. 21. In this way, we can capture this unusual word ordering elegantly in CCG.

**Fig. 22:**
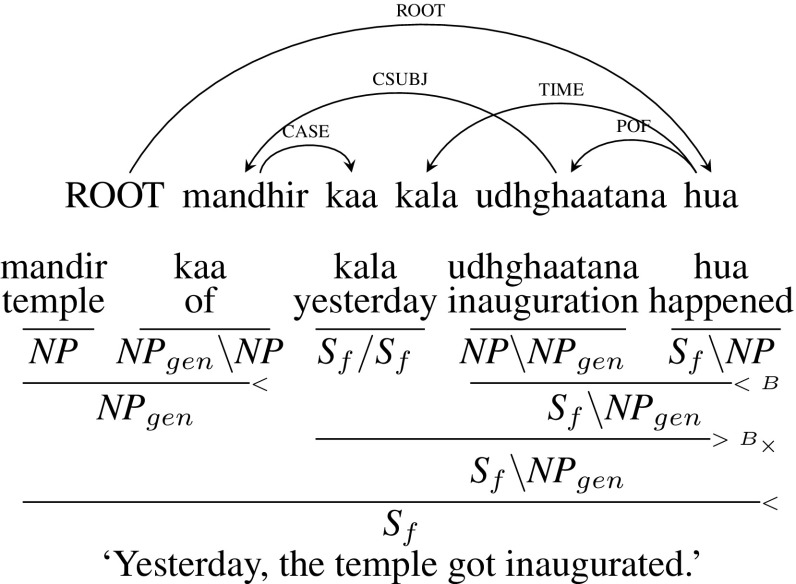
Dislocated/discontinuous genitives (time expression)

Hindi also has extensive use of “light” verbs, also called conjunct verbs. A conjunct verb is composed of a noun or an adjective followed by a verbalizer. Subject (SUBJ) or Object (OBJ) arguments of a conjunct verb can have the genitive case marker. In such cases, the arguments have a dependency relation with the noun of the conjunct verb since the agreement is with the noun of the conjunct verb and not with the verb. The free word-order nature of adverbs and time and/or place expressions can cause crossing arcs as in the following examples. Such constructions are called dislocated/discontinuous genitives. We treat Part-OF (POF) and subject/object of conjunct verb (CSUBJ/COBJ) as arguments. For example, in Fig. 22, the light verb *hua* (“happened”) looks for an *NP*, *udhghaatana* (“inauguration”) to its left. *udhghaatana* has a child *mandir kaa* (“of temple”) with CSUBJ dependency relation. Since CSUBJ is an argument relation, CCG category of *udhghaatana* is ${NP\setminus NP_{gen}}$ which looks for an *NP* with genitive marker to its left. *udhghaatana* first combines with the light verb *hua* and then with the optional time expression *kala* (“yesterday”) leading to ${S_{f}\setminus NP_{gen}}$. The verb phrase ${S_{f}\setminus NP_{gen}}$ is then combined with the noun phrase with genitive marker *mandir kaa* (“of temple”) resulting in a sentence ${S_{f}}$.

**Fig. 23:**
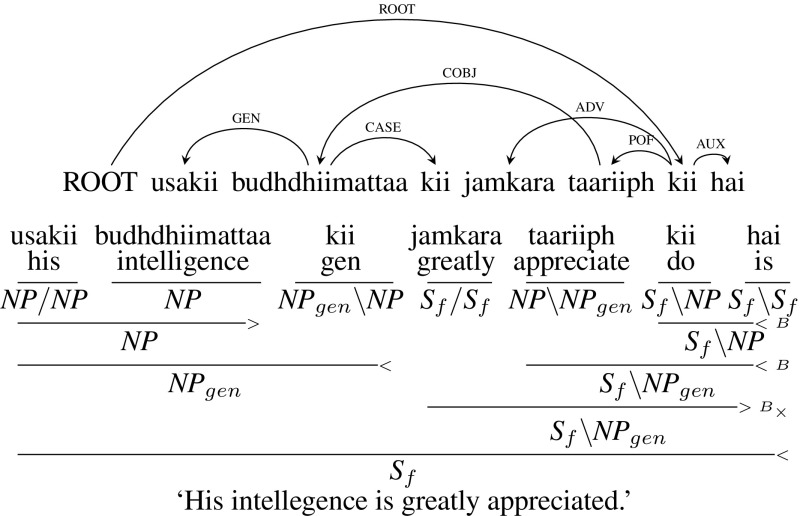
Dislocated/discontinuous genitives (adverb)

Figure 23 is similar to Fig. 22, except that the noun with genitive marker *budhdhiimattaa kii* (“intelligence”) is in COBJ dependency relation with the noun of the conjunct verb *taariiph* (“appreciate”). Also the intervening node *jamkara* (“greatly”) which is the cause for the crossing arc is an adverb (ADV) unlike the time expression in the previous case.

### Others

Other major dependency labels/constructions which lead to crossing dependency arcs are time/place expressions (TIME/PLACE), noun modifiers (NMOD), SUBJ. These labels corresponds to 9% of crossing arcs.

Similar to adverbs, time/place expressions, due to freer word-order nature of Hindi, can occur at any place in the sentence and can be handled using crossed composition in general cases. But, when these occur between nouns in genitive relation or in the conjunct verbs constructions (as in Sect. 8.5), they lead to crossing arcs, and are handled as discussed in Sect. 8.5.

NMOD is the label for noun modifier. NMOD constructions which lead to crossing arcs are similar to those of genitives as in Sect. 8.5. SUBJ constructions also engender crossing arcs similarly to the OBJ constructions/topicalization in Sect. 8.3. These constructions are handled similarly to the ones described in the previous sections.

## Analysis of the Hindi CCGbank

In this section, we provide a brief analysis of the different CCG categories and combinators in the Hindi CCGbank. Table 2 lists the top 12 most frequent CCG categories in both coarse-grained and fine-grained versions of the lexicon. The most common categories are the category for nouns (*NP*) and noun modifiers like adjectives and determiners (*NP* / *NP*). The next most frequent categories are the categories for post-position markers for nouns and auxiliary or tense, aspect and modality (TAM) markers for verbs. ${S_{f}\setminus S_{f}}$ and ${NP\setminus NP}$ are the categories for auxiliary or TAM markers for verbs and post-position markers for nouns respectively. The post-position marker of an adjunct noun phrase gets the category ${(S_{f}/ S_{f})\setminus NP}. {(NP/ NP)\setminus NP}$ is the category for both genitive marker and conjunction in NP coordination. ${(S_{f}\setminus NP)\setminus NP}$ and ${S_{f}\setminus NP}$ are the categories for transitive and intransitive verbs respectively. Adjectival phrase gets a category *JJP*. (*NP* / *NP*) / (*NP* / *NP*) is the category for modifier of a noun modifier and ${CCP/ S_{f}}$ is the category for subordinate conjunction.

Categories in the top 12 list of the fine-grained lexicon but not in the coarse-grained are *NP*[0], ${NP[0\_ne]\setminus NP}$ and ${NP[0\_ko]\setminus NP}$. In this lexicon, the coarse category for nouns gets split into *NP* (the category for a noun with a separate lexical item as a case marker) and *NP*[0] (the category for a noun without any case marker). For example, in noun chunks *raam ne* (“Ram ERG”) and *raam* (“Ram”), the category of *raam* is *NP* in first case and *NP*[0] in the later case. 0 here means that the case marker appeared as a separate lexical item. For example, *raam ne* (“Ram ERG”) will have NP[0_ne] as the category whereas *usne* (“he+ERG”) will have NP[ne] as the category. This is the notation followed in the Hindi dependency treebank. The remaining two categories, ${NP[0\_ne]\setminus NP}$ and ${NP[0\_ko]\setminus NP}$, are the categories for ergative (‘ne’) and dative (‘ko’) case-markers.

**Table 2:** Distribution of CCG categories in coarse-grained (left) and fine-grained (right) lexicon

CCG category	Percentage (%)	CCG category	Percentage (%)
*NP*	28.09	*NP*	17.67
*NP* / *NP*	16.45	*NP* / *NP*	16.44
${S_{f}\setminus S_{f}}$	9.05	*NP*[0]	9.11
${NP\setminus NP}$	6.99	${S_{f}\setminus S_{f}}$	9.05
${(S_{f}/ S_{f})\setminus NP}$	6.66	${(S_{f}/ S_{f})\setminus NP}$	5.91
${(NP/ NP)\setminus NP}$	4.53	${(NP/ NP)\setminus NP}$	4.09
${S_{f}/ S_{f}}$	2.56	${S_{f}/ S_{f}}$	2.56
${(S_{f}\setminus NP)\setminus NP}$	2.21	*JJP*	2.12
*JJP*	2.11	(*NP* / *NP*) / (*NP* / *NP*)	1.90
${S_{f}\setminus NP}$	2.05	${NP[0\_ne]\setminus NP}$	1.84
(*NP* / *NP*) / (*NP* / *NP*)	1.90	${S_{f}\setminus NP[0]}$	1.82
${CCP/ S_{f}}$	1.60	${NP[0\_ko]\setminus NP}$	1.77

Table 3 shows the distribution of different CCG combinators in the Hindi CCGbank. Since Hindi is a verb final language, the backward application and composition combinators are more frequent than forward application and composition combinators. Due to freer word-order nature and crossing dependency arcs, there are around 0.5% of crossed composition combinators in the Hindi CCGbank. This shows the importance of crossed composition combinators for freer word-order languages.

**Table 3:** Distribution of combinators in the Hindi CCGbank

CCG combinator	Percentage (%)
Forward application (>)	38.61
Backward application (<)	45.90
Forward composition (>B)	0.01
Backward composition (<B)	14.99
Forward crossed composition (${>}B_{X}$)	0.04
Backward crossed composition (${<}B_{X}$)	0.45

## Conclusion

We presented an approach for automatically creating a CCGbank from a dependency treebank for Hindi which is a morphologically rich, freer word-order and verb final language. We created two types of lexicon: fine-grained which keeps morphological information in noun categories and coarse-grained which doesn’t. We have provided a detailed analysis of various long-range dependencies like coordinate and relative constructions, and shown how to handle them in CCG. We have also discussed in detail the different word-orders that arise from the free word-order nature of Hindi in various constuctions, and provided a unified projective analysis for them under CCG. We have also provided a brief statistical analysis of the different CCG categories and combinators occurring in the Hindi CCGbank.

The approach described here has already been successfully applied to Telugu, another Indian language (Kumari and Rao 2015). In future we would like to extract CCG lexicons and/or CCGbanks for the many other languages for which dependency treebanks are available, including the languages of the CoNLL dependency parsing shared tasks (Buchholz and Marsi 2006; Nivre et al. 2007a) and universal dependency treebanks (McDonald et al. 2013).^19^ State of the art results for parsers trained and tested on the treebank are reported in Ambati et al. (2013, 2014); Ambati (2016).

## Figures and Tables

**Fig. 24:**
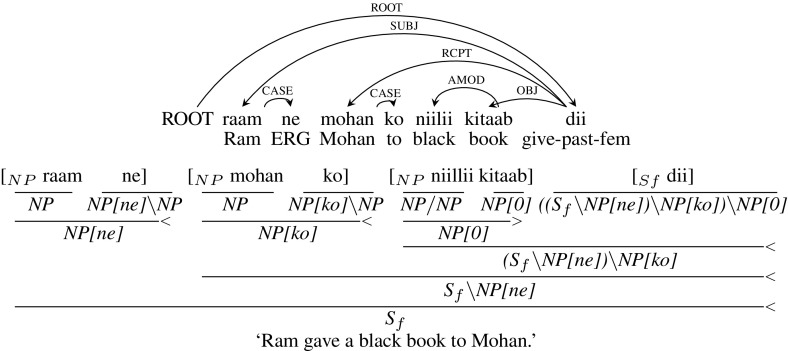
Example dependency tree and CCG derivation (fine-grained)

**Fig. 25:**
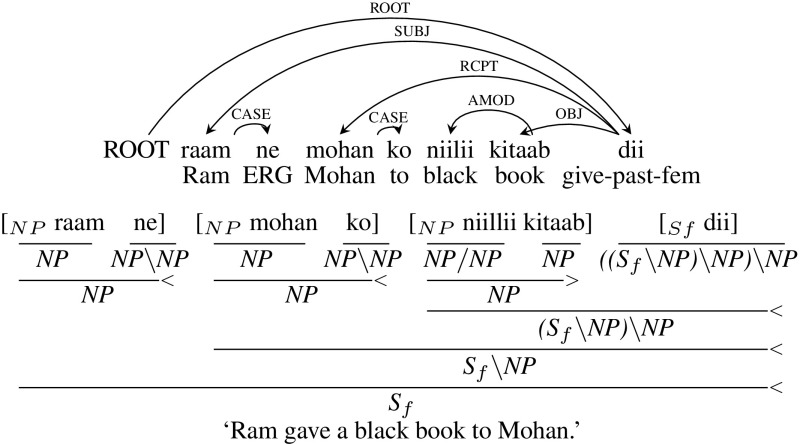
Example dependency tree and CCG derivation (coarse-grained)

## Appendix 1: Hindi dependency labels

See Table 4.

**Table 4:** Hindi dependency labels and their English equivalents

Hindi depenency label	English equivalent	Description
k1 (kartha)	SUBJ	Subject/agent
k1s (kartha samanadhikarana)	SCOM	Noun complements of kartha
k2 (karma)	OBJ	Object/patient
k3 (karana)	INST	Instrument
k4 (sampradaana)	RCPT	Recipient
k5 (apaadaana)	SRC	Source
k7t (kaalaadhikarana)	TIME	Time expression
k7p (deshadhikarana)	PLACE	Place expression
r6 (shashthi)	GEN	Possessive/genitive marker
nmod_relc	RELC	Relative clause
vmod	VMOD	Verbal modifier
nmod	NMOD	Noun modifier
nmod__adj	AMOD	Adjectival modifier of a noun
lwg__psp	CASE	Case marker
lwg__aux	AUX	Auxiliary verb or tense, aspect and modality marker for verb
pof	POF	Part-OF units such as conjunct verbs
rs	CCOM	Clausal complement
r6–k1	CSUB	SUBJ of conjunct verb
r6–k2	COBJ	OBJ of conjunct verb

## Appendix 2: Hindi chunk tags

See Table 5.

**Table 5:** Hindi Chunk tagset

S. No	Chunk type	Tag name
1	Noun chunk	NP
2.1	Finite verb chunk	VGF
2.2	Non-finite verb chunk	VGNF
2.3	Infinitival verb chunk	VGINF
2.4	Verb chunk (gerund)	VGNN
3	Adjectival chunk	JJP
4	Adverb chunk	RBP
5	Chunk for negatives	NEGP
6	Conjuncts	CCP
7	Chunk fragments	FRAGP
8	Miscellaneous	BLK

## Appendix 3: Machine-readable format

CCG derivation for the first sentence in the Hindi dependency treebank guidelines using fine-grained lexicon is given below. We follow the format of Hockenmaier and Steedman (2007) for representing the binary CCG derivation trees with the bracketed notation.

There are two types of nodes in the derivation trees: Leaf nodes and Non-leaf nodes. Leaf nodes have six fields.

<L NP[ne] NNP NNP raam NP[ne]>

<L CCGCat mod-POS-tag orig-POS-tag word CCGCat2>

L represents that it is a leaf node. CCGCat is the CCG category of the node. Unlike English, POS tag is not modified during the conversion of dependency trees to CCG derivations. So, in Hindi CCGbank, mod-POS-tag and orig-POS-tag both represent the POS tag of the word. Lexical item is represented using word field. In English CCGbank, CCGCat2 slot is used to represent predicate-argument structure of the CCG category. In Hindi CCGbank, we just use the lexical CCG category to fill this slot.

Non-leaf nodes have four fields. T represents that the node is a non-leaf node. CCGCat is the CCG category of the node. head takes two values: 0 if the left node is the head and 1 if the right node is the head. Since the CCG derivation trees are binary trees, children field will have 1 or 2 based on whether there are one or two children. Example non-leaf node is given below.

<T NP[ne] 0 2

<T CCGCat head children

CCG derivation tree with coarse-grained lexicon is provided below in machine-readable format along with the dependency tree and derivation (Fig. 25). Figure 24 presents the CCG derivation of the same sentence using fine-grained lexicon.

## Footnotes

1
http://www.di.unito.it/~tutreeb/.

2
http://www.ethnologue.com/statistics/size.

3 S = Subject; IO = Indirect Object; DO = Direct Object; V = Verb; ERG = Ergative; DAT = Dative.

4 Hindi is split-ergative. The ergative marker appears on the subject of a transitive verb with perfect morphology.

5 All examples have been taken from the corpus, although in many case they are simplified by the omission of modifiers and conjunction.

6
http://nextens.uvt.nl/depparse-wiki/DataFormat.

7 We return below to the question of case marking and agreement.

8 We treated CASE in this manner for the case of consistency with the dependency treebank and leave more linguistically sophisticated treatments of CASE for future work (although see Sect. 5.1 for a type-raising analysis).

9 VGF is the chunk tag for finite verb chunk in the Hindi dependency treebank. But for the sake of brevity we use S$_{f}$ notation here. A list of the Hindi chunk tags are provided in the “Appendix 2”.

10
http://openccg.sourceforge.net/.

11 This treatment constitutes a slight difference from English CCGbank, where coordination is treated syncategorematically, with conjunction bearing the category *conj*.

12 TV is the short form for ${((S_{f}\setminus NP)\setminus NP)}$, the transitive verb category.

13 We are not handling argument cluster coordination in the current version of the CCGbank since the current version doesn’t include unary type-changing rules. We will handle these constructions in the next version.

14 See Clark and Curran (2007) for details on how indexed categories are used to extract dependencies.

15 It is easy to re-construct the original dependency with the help of lexical item *yaha* (“this”). We can find the parent of *ki* (“that”) and extract the lexical item *yaha* (“this”) from its sub-tree. Assigning it as the parent of *ki* (“that”) would result in the original dependency tree.

16 In Hindi dependency treebank POF (part-of) dependency label is used to represent part of units such as conjunct verbs.

17 For example, in Fig. 16, CCG derivation gives the dependency between *hai* (“is”) of relative clause and *hai* (“is”) of main clause. As the chunk with *vo* (“that”) root word (here *vaha*) is *vaha ladakaa* (“that boy”), the head of *hai* (“is”) as per Hindi dependency guidelines would be *ladakaa* (“boy”).

18 Indices for categories are not part of the lexicon but indices are used while extracting dependencies from the CCG derivation.

19
http://universaldependencies.org/.
